# Lactic Acid Bacteria as In Vivo Protective Strategy Against Dietary Methylmercury Exposure

**DOI:** 10.3390/jox16030107

**Published:** 2026-06-08

**Authors:** Luzmila Burbano, Pilar Rodríguez-Viso, Manuel Zúñiga, Vicente Monedero, Vicenta Devesa, Dinoraz Vélez

**Affiliations:** Instituto de Agroquímica y Tecnología de Alimentos (IATA.CSIC), Calle Agustín Escardino 7, 46980 Paterna, Spain; luzmila.burbano@utm.edu.ec (L.B.);

**Keywords:** inorganic mercury, methylmercury, lactic acid bacteria, dietary strategies, toxicokinetics, tissue bioaccumulation, food, mice, rats

## Abstract

The aim of this study was to characterize the toxicokinetics of dietary Hg and to identify lactic acid bacteria (LAB) strains capable of reducing methylmercury (MeHg) accumulation in organs. BALB/c mice and Wistar rats were exposed via drinking water [Hg(II) MeHg] and feed (swordfish, mushrooms). The data showed that the feed matrix did not modify MeHg accumulation, though it reduced Hg(II) accumulation compared to the data observed with drinking water. Two in vitro LAB strains (*L. intestinalis* LE1 and *L. johnsonii* LE2) were selected to determine their efficacy in reducing tissue accumulation in mice exposed for 40 days to dietary MeHg. Daily dosing of both strains produced a relevant reduction in Hg content in the organs of animals exposed through drinking water (18–64%). The LE1 strain also reduced Hg content in animals exposed via feed (13–40%). LAB could be a useful strategy in populations which are chronically exposed to Hg through their diet.

## 1. Introduction

The primary source of exposure to mercury (Hg) in the general population is through diet, where the metal is predominantly present in the form of methylmercury (MeHg). Large marine predatory fish (swordfish, shark, bonito and tuna, among others) are the main contributors to Hg intake. According to the report of the Joint FAO/WHO Expert Committee on Food Additives (JECFA), among 6114 fish samples collected from different countries approximately 5% exceeded 1 mg/kg of Hg which is the maximum level of this metal permitted for certain fish species under Commission Regulation (EU) 2023/915 [1]. Inorganic mercury, mainly in the form of Hg(II), is not a major contaminant in the diet; its primary sources include certain terrestrial foods, such as mushrooms [2,3] and some fishery products [4]. The JECFA has established a tolerable weekly intake (TWI) of 1.6 μg/kg of body weight (bw) for MeHg and 4 µg/kg bw for inorganic Hg [1]. A warning has been issued by the European Food Safety Authority (EFSA) regarding the potential risks associated with the consumption of fishery products for certain population groups in Europe [4]. This concern arises due to the fact that the 95th percentile of weekly Hg intake is either close to or surpasses the TWI across all age groups. Moreover, frequent consumers of fishery products may exceed this reference value by up to 6 times [4].

Some studies have been carried out to assess the risk associated with the regular consumption of fishery products by measuring biomarkers of exposure such as Hg contents in hair and blood. Sheehan et al. [5], in a review on Hg exposure, reported the highest Hg values in hair and blood to be found in populations with diets based on fishery products. These authors highlighted the Hg values recorded in coastal populations of Southeast Asia, the Western Pacific, and the Mediterranean, with average levels close to or above the reference values proposed by the United States Environmental Protection Agency (EPA) (1 mg/kg in hair; 5.8 μg/L in blood) [6,7]. Moreover, areas at risk from artisanal mining have been identified. In some communities of the Amazonian region, the Hg contents greatly exceed reference values [8,9,10]. In general, in these mining areas, local fish—a major component of the population’s diet—have high concentrations of MeHg [10,11].

Several studies have evaluated the toxicokinetics of Hg(II) and MeHg using different dosage forms. However, few studies have used naturally contaminated food to deliver the toxic metal [12,13]. A more comprehensive understanding of the absorption, distribution, accumulation, and excretion of Hg(II) and MeHg through contaminated food is necessary to adequately characterize dietary exposure and be able to design strategies to reduce the associated risk. Health and food safety agencies in several countries recommend limiting the consumption of certain fish products in susceptible populations [14,15]. However, the EFSA warns that due to the high nutritional value of some of these products, a general recommendation to avoid consumption is not the most appropriate measure to reduce Hg exposure [16]. Among the available options, recent studies have explored the possibility of modulating the passage of the toxic element into the systemic circulation following its intake (bioavailability). Previous results have shown that some strains of lactic acid bacteria (LAB) are able to reduce the amount of MeHg that is available after in vitro simulated gastrointestinal digestion (bioaccessibility) [17]. Furthermore, certain strategies within this framework have demonstrated the efficacy of selected LAB strains in reducing the transport of MeHg through the intestinal epithelium, as evidenced in cell culture models [18]. In turn, Majlesi et al. [19] have evidenced a decrease in metal accumulation in the liver and kidneys after dosing rats by gavage with Hg(II) (10 mg/L) together with *Bacillus coagulans* or *Lactiplantibacillus plantarum* (1 × 10^9^ viable bacterial cells/mL) for 48 days. However, there are no studies assessing the protective effect against exposure to MeHg, the most common mercury form in the diet.

The present study was carried out to characterize the toxicokinetics of Hg consumed through water and naturally contaminated food, and to evaluate the effectiveness of several LAB strains in reducing the passage of MeHg through the intestinal epithelium and its accumulation in target organs, using experimental animals dosed sub-chronically through water and food. The novelty of this study lies in considering the diet as a key exposure route and in evaluating LAB-based approaches to reduce the absorption and accumulation of food-borne MeHg, instead of relying on simplified aqueous exposure models.

## 2. Materials and Methods

### 2.1. Mercurial Species

The solutions of Hg(II) and MeHg used in this study were prepared through the dilution of the commercial-standard Hg(NO_3_)_2_ (1000 mg/L, Merck, Barcelona, Spain) and CH_3_HgCl (1000 mg/L, Alfa Aesar, Barcelona, Spain), respectively.

### 2.2. Animal Model

Female BALB/c mice and Wistar rats (Envigo RMS, Barcelona, Spain) were used for the in vivo study. The animals were acquired at 8 weeks of age and weighed between 16.2 and 18.7 g for mice and between 241 and 267 g for rats. Throughout the study, animals were kept under controlled environmental conditions (12 h light/dark cycle, 22 ± 1 °C, 75 ± 5% humidity) in the facilities of the Animal Production and Experimentation Section of the Central Support Service for Experimental Research (SCSIE) of the University of Valencia (Spain). The animals fed *ad libitum* on standard rodent maintenance feed with a low Hg concentration (<0.01 ng/g). The experimental procedures were designed in accordance with the European Union Directive 2010/63/EU, presented according to the ARRIVE guidelines for reporting animal research, and approved by the Agriculture, Fisheries, and Food Council of the Valencian Government (Spain).

### 2.3. Characterization of the Toxicokinetics of Hg Administered Through Water or Food

Two types of assays were performed: single exposure to determine the entry of Hg into the blood and its disappearance, and long-term exposure to assess tissue accumulation and fecal excretion. In the single-dose assay, aqueous standard solutions of Hg(II) (0.5 mg/kg bw) or MeHg (0.25 mg/kg bw) were administered by gavage to mice (*n* = 5) and rats (*n* = 3), and blood samples were obtained at different times (up to 24 h for mice and up to 4 days for rats). Total Hg contents in blood were determined by microwave digestion and cold vapor atomic fluorescence spectrometry (CV-AFS) (Section 2.5).

In the long-term studies, the animals were randomized into four groups, with three animals per group for rats and fiver per group for mice. Two groups were exposed to 5 mg/L Hg(II) or 2.5 mg/L MeHg via drinking water for 20 days. The remaining two groups were exposed to Hg through their diet for 20 days, using feed enriched with swordfish or mushroom. For feed formulation, samples of freeze-dried swordfish [6.9–8.5 mg/kg Hg dry weight, dw] or dried mushroom [4.7–5.2 mg/kg dw Hg] were mixed with maintenance feed. Table 1 shows the final consumption data per body weight for each rodent.

During exposure, body weight, the consumption of water and food, as well as the general health conditions and behavior of the experimental animals were monitored weekly. At the end of exposure, the animals were sacrificed by isoflurane inhalation and cervical dislocation, and samples of small intestine, colon, liver, kidney, brain, and blood were collected. In addition, at various intervals during the treatment, fecal samples were obtained for the determination of Hg fecal excretion and the degree of MeHg demethylation (Section 2.5).

### 2.4. Effect of Lactic Acid Bacteria Strains on the Toxicokinetics of MeHg

#### 2.4.1. Isolation and Identification of LAB Strains from Mice Fecal Samples

Fecal pellets from BALB/c mice fed with standard rodent maintenance feed were collected and stored at −80 °C until use. Samples were suspended 1/10 (*w*/*v*) in phosphate buffered saline (PBS, Hyclone, Merck, Barcelona, Spain) and plated in De Man, Rogosa, and Sharpe (MRS) (BD Difco, Merck, Barcelona, Spain) agar plates. Plates were incubated at 37 °C for 48 h. Randomly selected colonies which differed in their morphological characteristics were taken and isolated by repeated streaking on MRS agar plates. For storage, cultures were grown in MRS broth at 37 °C for 24 h, glycerol was added at 15% (*v*/*v*) final concentration, and 2 mL aliquots were maintained at −80 °C until use. The preliminary identification of isolates was carried out by PCR amplification and the partial sequencing of 16S rRNA gene with primers 27F (AGAGTTTGATCCTGGCTCAG) and 1492R (GGTTACCTTGTTACG ACTT) [20]. Homology searches were performed in the NCBI database by BLAST 2.17.0 version (https://blast.ncbi.nlm.nih.gov/Blast.cgi, accessed on 1 May 2026). Six isolates were selected for subsequent assays.

#### 2.4.2. In Vitro Screening of Bacterial Strains

The LAB strains used throughout the study were: *Lactobacillus intestinalis* (LE1), *Lactobacillus johnsonii* (LE2), *Ligilactobacillus murinus* (LE3), *Lactococcus* sp. (LE4), *L. murinus* (LE5), and *L. murinus* (LE6). Bacteria were routinely grown in MRS at 37 °C under static conditions. Bacterial cells were harvested by centrifugation (4000× *g*, 10 min), washed with one volume of PBS, and centrifuged under the same conditions.

To determine the MeHg binding capacity of the isolated strains, cells were suspended at an OD_600_ of 4 in a solution of MeHg (1 mg/L) in PBS and incubated at 37 °C for 2 h. Then, the samples were centrifuged at 4000× *g* for 5 min at 4 °C and Hg concentrations in the supernatant and the cell pellet were analyzed by microwave digestion CV-AFS following the procedure described in Section 2.5.

The Hg binding capacity of the LAB strains was also analyzed under gastrointestinal digestion conditions, following the protocol described by Jadán-Piedra, Alcántara, Monedero, Zúñiga, Vélez and Devesa [17] with some modifications. Cooked swordfish (0.5 g, concentration: 2.2 ± 0.2 mg MeHg/kg) or a standard solution of MeHg (1 mg/L) with or without the LAB strains (final OD_600_ of 4) were weighed, and 20 g of deionized water was added. The pH of the sample was adjusted to 2.0 with 6 mol/L HCl. For the gastric stage, porcine pepsin (Sigma, Barcelona, Spain, enzymatic activity 944 U/mg protein) at 2% (*w*/*v*) in 0.1 mol/L HCl was added to the sample with 0.1 mg pepsin/g of solution. The mixture was incubated at 37 °C for 2 h with constant shaking (400 strokes/min). The digest was cooled to room temperature and the pH was adjusted to 6.5 with NH_3_ (Scharlau, Scharlab, Barcelona, Spain). A solution of porcine pancreatin (Sigma, activity equivalent to 4× US Pharmacopeia specifications/mg pancreatin) 0.4% (*m*/*v*) and taurocholic acid (Alpha Aesar, Barcelona, Spain) 4.8 mM in 0.8 mM (NH_4_)_2_CO_3_ (Merck) was added to the digest to provide 0.02 mg pancreatin/g solution and 24 µM taurocholic acid. The mixture was incubated at 37 °C for 2 h with constant shaking (400 strokes/min). After the simulated digestion, samples were centrifuged (15,300× *g*, 4 °C, 30 min) and total Hg was quantified in the soluble fraction obtained (bioaccessible fraction) following the protocol described in Section 2.5.

#### 2.4.3. In Vivo Evaluation of the Efficiency of LAB in Reducing Mercury Exposure

On the basis of the results of the in vitro assays, two strains were selected for testing in laboratory mice: *L. intestinalis* (LE1) and *L. johnsonii* (LE2). Animals (*n* = 42) were housed separately in 6 groups of 7 mice. A preconditioning period with LAB was performed in which four groups received a daily bacterial dose (1 × 10^9^ CFU in 50 µL PBS) by oral gavage for a total of 8 days, and the remaining two groups (controls) were given 50 µL of PBS by oral gavage. After this period, all the animals were exposed to MeHg sub-chronically during 40 days via drinking water (5 mg/L) (dose of 0.50 mg/kg bw/day) or feed enriched with swordfish (1.8 mg/kg final concentration of the feed pellet; dose of 0.25 mg/kg bw/day). Thus, there were 6 groups with the following treatments (Table S1, Supplementary Data): MeHg through drinking water (control A); MeHg in drinking water and a daily gavage dose of LE1 and LE2 (groups B and C); MeHg through feed (Control D); MeHg through feed and a daily gavage dose of LE1 and LE2 (groups E and F). At the end of the experiment, the animals were euthanized and liver, small intestine, colon, blood, brain, and kidneys were collected for determining Hg accumulation. Mercury quantification was performed by microwave digestion CV-AFS (Section 2.5).

### 2.5. Determination of Total and Inorganic Hg

To determine total Hg, samples were digested using an accelerated microwave digestion system (MARS, CEM, Alenium, Barcelona, Spain). The samples were placed in microwave vessels, and 4 mL of 14 mol/L HNO_3_ (Merck) and 1 mL of H_2_O_2_ (30%, Prolabo, VWR, Barcelona, Spain) were added. The vessels were irradiated (180 °C, 15 min) and the resulting digest was allowed to stand for 12 h to remove nitrous vapors. Subsequently, they were brought to final volume with 0.6 mol/L HCl and quantified by CV-AFS (PSA 10.025 Millennium Merlin, PS Analytical, Microbeam, Barcelona, Spain). For the determination of inorganic Hg in feces, samples (0.05–0.2 g) with 5 mL of HCl 2 mol/L (*v*/*v*, Merck) were placed in microwave vessels and irradiated at 100 °C for 20 min. Subsequently, they were brought to final volume with deionized water.

The analytical conditions of the CV-AFS detection method were as follows: reducing agent, 2% (*w*/*v*) SnCl_2_ (Scharlab, Scharlab, Barcelona, Spain) in 1.8 mol/L HCl at a flow rate of 4.5 mL/min; carrier solution, 0.6 mol/L HCl at a flow rate of 9 mL/min; carrier gas, Ar at a flow rate of 0.3 L/min; drying gas, air at a flow rate of 2.5 L/min; specific Hg lamp (PSAnalytical); 254 nm filter. Quantification was performed against an external calibration curve (0.1–2 ng/mL). Quality control of the quantification was assessed by analysis of a reference water sample (RTC, QCI-049-1, LGC Standards, Barcelona, Spain) with a certified Hg content of 40.8 ± 0.396 µg/L.

### 2.6. Statistical Analysis

A Student’s *t*-test or one-way analysis of variance (ANOVA) with multiple *post hoc* comparisons (Fisher HSD test) were employed when the requirements for normality (Shapiro–Wilk test) and the homogeneity of variances between all groups (Brown–Forsythe test) were met. In the rest of the cases, data were analyzed applying the Mann–Whitney U-test or the Kruskal–Wallis test with multiple comparisons using the Dunn test. Differences were considered statistically significant at *p* < 0.05. The analyses were performed using SigmaPlot 14.5 (Systat Software Inc.,Grafiti LLC, Palo Alto, CA, USA). The GPower 3.1 application was employed to determine the sample size needed to perform the in vivo analyses (α = 0.05, 1 − β = 0.8). It should be acknowledged that the number of rats used in the toxicokinetic experiments (*n* = 3 per group) is relatively limited. Although this design is commonly used in exploratory studies of toxicokinetics and tissue distribution [21,22], it may reduce the statistical power of the analysis.

## 3. Results

### 3.1. Toxicokinetics of Hg Present in Water and Food

**Figure 1:**
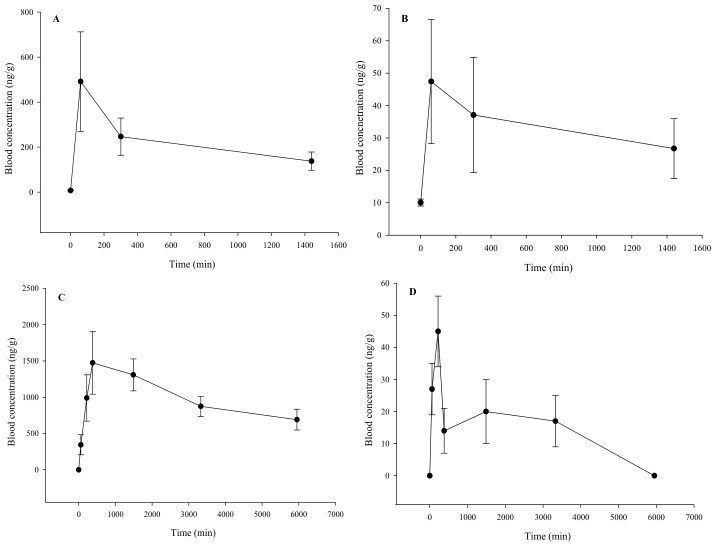
Total Hg concentration in blood after administration by gavage of Hg(II) (0.5 mg/kg bw) and MeHg (0.25 mg/kg bw) in mice ((**A**,**B**) respectively) and rats ((**C**,**D**) respectively) at different times. Values expressed as ng/g blood (mean ± SD, *n* = 3–5).

Wistar rats and BALB/c mice were administered a single dose of Hg(II) (0.5 mg/kg bw) or MeHg (0.25 mg/kg bw) to determine the appearance of Hg in blood and its half-life. The data obtained showed the maximum concentration of Hg(II) in blood to be lower (Cmax mice: 47.4 ng/g; Cmax rats: 45 ng/g) than that observed for MeHg (Cmax mice: 491.5 ng/g; Cmax rats: 1474 ng/g). In mice, the appearance of Hg in blood was relatively fast (tmax: 60 min), and most of the Hg disappeared 24 h after dosing (Figure 1A,B). In rats, the peak concentration in blood was reached after 420 min, and the Hg clearance rate was lower. It took a minimum of four days to reach levels comparable to those recorded immediately before dosing for Hg(II) (Figure 1D). In contrast, elevated levels of Hg persisted even after four days following the administration of MeHg (Figure 1C).

**Figure 2:**
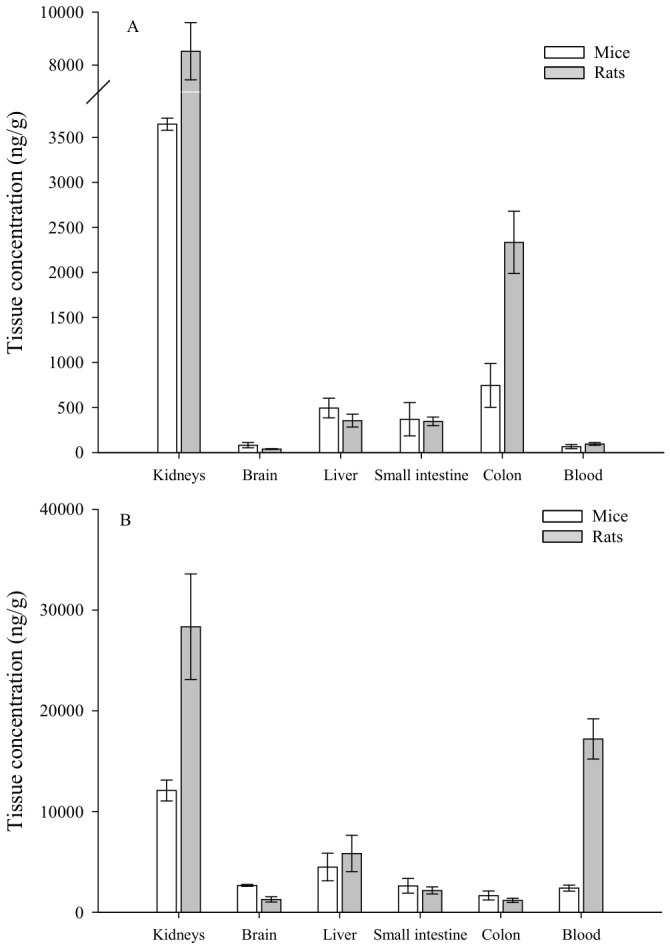
Total Hg concentration in different organs and blood after exposure for 20 days to Hg(II) (**A**) or MeHg (**B**) through drinking water. Values expressed as ng/g tissue (mean ± SD, *n* = 3–5).

Mercury contents in various organs of the animals exposed for 20 days to Hg(II) (0.5 mg/kg bw) or MeHg (0.25 mg/kg bw) through drinking water are shown in Figure 2A,B, respectively. In general, animals exposed to Hg(II) showed a lesser accumulation compared to those exposed to MeHg, even though the concentration of the inorganic form dosed through drinking water was higher. After exposure to Hg(II), the greatest accumulation of Hg was observed in the kidneys of both species. However, the distribution in organs varied depending on the type of rodent. The accumulation of Hg in the colon of rats was much higher than in the small intestine and liver. In mice, although the colon also showed greater accumulation, Hg accumulation did not differ significantly from the contents found in the liver and small intestine. In both rodent species, Hg contents in the brain and blood were low. After exposure to MeHg, the greatest concentration of Hg was observed in the kidneys. Accumulation of Hg also varied depending on the type of rodent. In mice, Hg contents in blood were very low, and similar to those observed in both portions of the intestine; however, in rats, the presence of Hg in the blood was very high.

**Figure 3:**
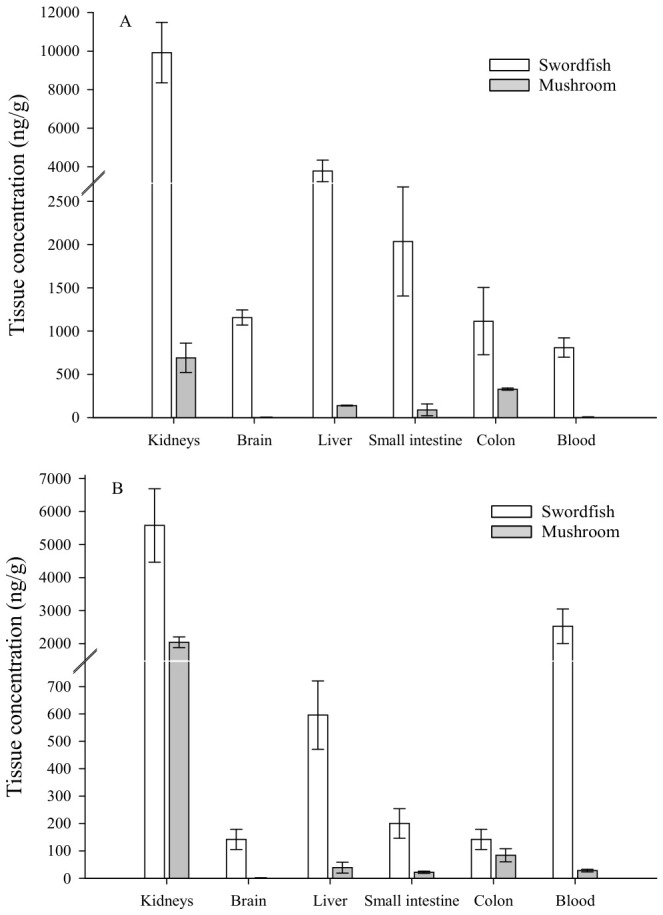
Total Hg concentration in different organs and blood in mice (**A**) and rats (**B**) exposed for 20 days to feed enriched with swordfish or mushrooms. Values expressed as ng/g tissue (mean ± SD, *n*= 3–5).

The accumulation of Hg in the organs of mice (Figure 3A) and rats (Figure 3B) exposed to Hg via feed showed a similar distribution to that in animals exposed via drinking water. Animals exposed to MeHg (feed prepared with swordfish) showed a greater accumulation in the organs, and only the kidneys showed the relevant accumulations in animals exposed to inorganic Hg (feed prepared with mushrooms). Once again, a remarkable accumulation of Hg in the blood was observed in rats fed MeHg-rich feed.

Table 2 shows the fecal contents of total and inorganic Hg 17 days after the onset of exposure. The fecal excretion of Hg was greater after the administration of Hg(II) than MeHg through drinking water. These differences, however, were less pronounced when Hg was administered via feed. Speciation data showed a high degree of demethylation of MeHg regardless of the rodent species and route of administration involved. Most of the Hg excreted in feces in animals dosed with MeHg via water or feed was in the inorganic form (60–83%).

### 3.2. Evaluation of Efficiency of LAB Strains in Reducing Dietary Hg Exposure

Considering the significantly lower bioavailability and tissue accumulation of Hg(II) compared to MeHg, and the prevalence of this latter form in food, we decided to explore strategies aimed at mitigating exposure to MeHg. The mouse was selected as the animal model due to the high retention of MeHg in the blood of rats, making the toxicokinetics of mice more similar to those observed in humans [23].

#### 3.2.1. Evaluation of MeHg Uptake by LAB Strains

**Figure 4:**
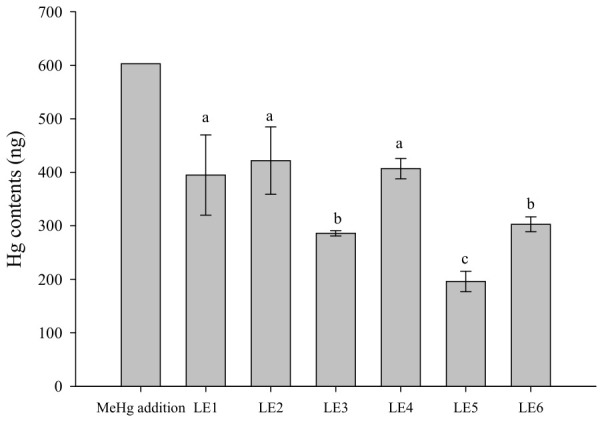
Retention of Hg by LAB strains incubated with a standard solution of MeHg (603 ng) for 2 h at 37 °C. Values expressed as ng Hg (mean ± SD, *n* = 3). Different letters show statistically significant differences between strains (*p* < 0.05).

In vitro screening assays were carried out with LAB, isolated from mice intestines, to select the most appropriate strains to carry out in vivo studies based on MeHg retention capabilities under different conditions. Figure 4 shows the amount of Hg retained by LAB strains after 2 h of exposure at 37 °C in a solution containing MeHg. The data show the following order in the degree of bacterial accumulation: strain LE2 > strain LE4 ≈ strain LE1 > strain LE6 ≈ strain LE3 > strain LE5. For the most efficient strains, the percentage of Hg retention compared to the initial amount added to the medium (603 ng of Hg) exceeded 50% (strain LE2: 72 ± 14%; strain LE4: 67 ± 3%; strain LE1: 65 ± 13%).

#### 3.2.2. Binding Capacity Under Gastrointestinal Digestion Conditions

**Figure 5:**
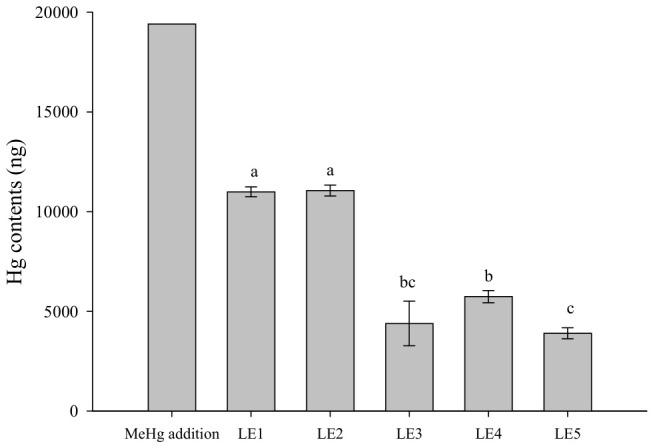
Retention of MeHg by bacteria during gastrointestinal digestion of standard solution of MeHg (20,000 ng). Values expressed as ng Hg (mean ± SD, *n* = 3). Different letters show statistically significant differences between strains (*p* < 0.05).

The bacterial retention of Hg was lower during the simulated gastrointestinal digestion of aqueous MeHg standards in the presence of LAB strains (21–57%, Figure 5), although it remained sufficiently high to consider some of these strains as suitable strategies for reducing Hg exposure in vivo.

**Figure 6:**
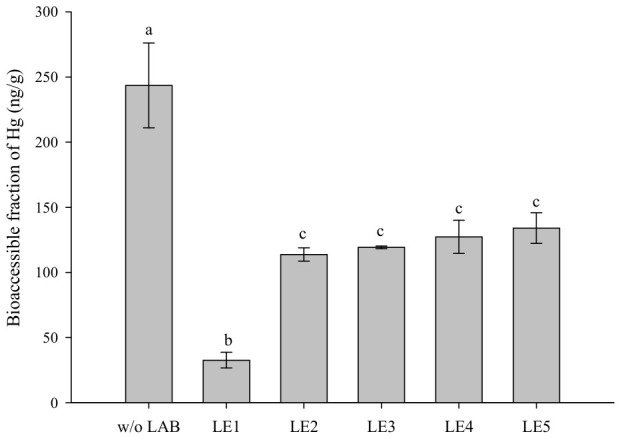
Total Hg bioaccessible fraction obtained after digestion of swordfish in absence or presence of different LAB strains. Values expressed as ng Hg/g food (mean ± SD, *n* = 3). Different letters show statistically significant differences between groups (*p* < 0.05).

Bacterial retention was also notorious when MeHg was present in a contaminated food matrix. Figure 6 shows the fraction of soluble Hg (bioaccessible fraction) after the digestion of MeHg-contaminated swordfish in the presence or absence of LAB strains. All the bacteria tested reduced the amount of Hg which was solubilized during digestion and therefore available for intestinal absorption (40–89%). The reduction produced by strain LE1 (87 ± 3%) was noteworthy.

#### 3.2.3. In Vivo Effect of LAB on the Toxicokinetics of MeHg in Food and Water

**Figure 7:**
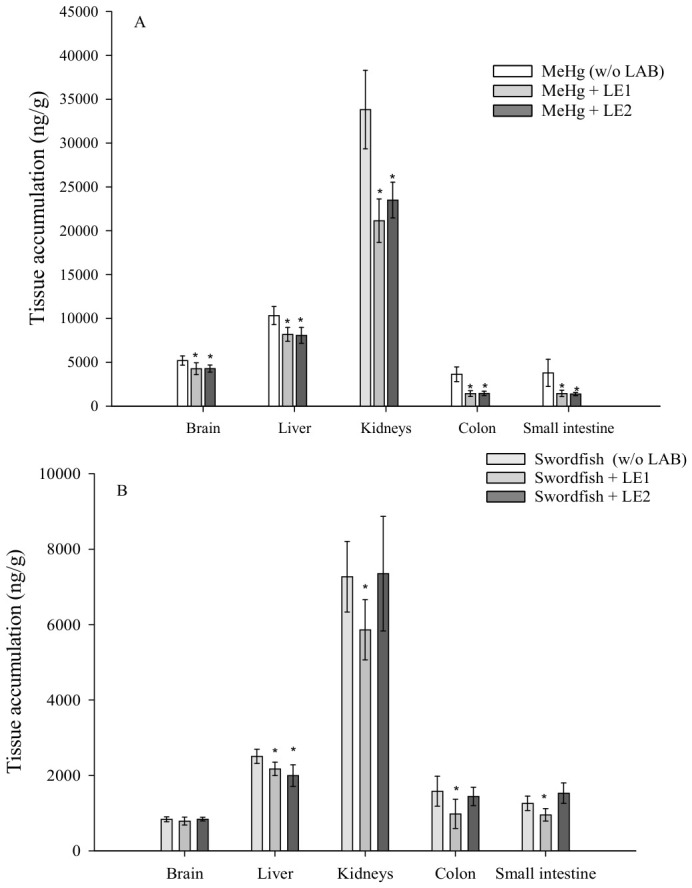
Total Hg concentration in different organs of mice exposed for 40 days to MeHg via drinking water (**A**) and food (**B**) with or without co-administration of LE1 and LE2. Values expressed as ng/g (mean ± SD, *n* = 7–9). Asterisks indicate statistically significant differences with respect to animals non-treated with LAB strains (*p* < 0.05).

Two LAB strains with a high MeHg retention activity (LE1 and LE2) were selected to test their potential to reduce Hg accumulation in mice after subchronic MeHg exposure via drinking water and a daily oral dose of the bacteria (Figure 7A). The strains used in this assay significantly reduced Hg concentrations in all the tissues tested. The reductions were more evident in the intestine (LE1: 62%; LE2: 64%) than in the kidneys (LE1: 37%; LE2: 31%), the liver (LE1: 22%; LE2: 22%) and the brain (LE1 and LE2: 18%).

In animals dosed through contaminated feed (Figure 7B), the reductions were smaller and were produced mainly by strain LE1 (liver: 13%; kidneys: 19%; colon: 24%; small intestine: 40%). Strain LE2 only reduced liver accumulation (30%) (Figure 7B).

## 4. Discussion

A comparative study of the toxicokinetics of the two major dietary forms of mercury [Hg(II) and MeHg] was made using different methods of administration (water and food) and two rodent models. Current studies on the absorption, distribution, accumulation, and excretion of Hg have mainly focused on assessing dosages through drinking water, which does not reflect the real-life situation, since Hg exposure is primarily through food consumption [4]. The single-dose trials conducted in this study show significant differences between both rodent species and both forms of Hg. As reported by other studies, the absorption of MeHg is higher [24]. Furthermore, there is a greater retention of MeHg in the blood of rats compared to mice (Figure 1). The red blood cell-to-plasma ratio presents large differences between species. A high ratio of about 300 was found in rats [23], while a ratio of 21, approximately, has been determined in humans [25], similar to the ratio (10) reported for mice [23]. Several studies have also demonstrated the greater affinity of MeHg for rat hemoglobin compared to that of mice or humans [26], accounting for a lower release rate and slower excretion.

The greater tissue accumulation of MeHg compared to Hg(II)—a phenomenon previously reported for animals dosed through water [27,28]—is also observed when food constitutes the dietary source (Figure 3). The distribution profiles of both species are also similar for both types of dietary administration. In all cases, there is a predominant accumulation in the kidney, as pointed out in previous studies [27,29]. Animals exposed to Hg(II) also show significant contents in the two intestinal segments analyzed, especially in the colon, possibly as a consequence of its low absorption. This pattern is similar in rats and mice. In the case of MeHg, the distribution profiles differ between the two rodent species due, as mentioned previously, to the significant retention of this Hg species in the blood of rats. This effect occurs in animals dosed with water and in those exposed through contaminated food.

In mice, the doses administered through water and food were similar, allowing for more precise toxicokinetic comparisons between the administration methods. In general, the magnitude of MeHg accumulation was similar in animals exposed through both routes. It should be noted that the forms in which MeHg is initially present in water and food are different. In the water solution, MeHg is present in the form of chloride, whereas in swordfish, MeHg has been shown to be predominantly bound to thiol groups of amino acids, peptides, and proteins [30,31]. In vitro studies have revealed that the mechanism of transport for both mercurial forms differs. The thiol-bound forms are transported through membrane transporters via molecular mimicry [32], whereas MeHgCl, due to its lipophilic nature, can be transported through simple diffusion [33]. However, this difference in transport mechanism does not seem to result in differences in the degree of absorption in animals. Mori et al. [34] did not observe significant differences in Hg concentrations in plasma or blood in rats administered orally with either MeHgCl or MeHgCys. Moreover, Bourdineaud, Marumoto, Yasutake, and Fujimura [13] reported similar tissue accumulations in mice fed with feed spiked with a MeHgCl solution and mice fed with feed prepared with lyophilized fish contaminated with the same concentration of MeHg. Some studies have suggested that, due to the high content of thiol-containing molecules in the intestinal lumen and the high reactivity of Hg with thiols, ingested MeHg may rapidly combine with them to form various conjugates, which are the primary absorbable forms, regardless of its initial form in water or food [35]. This could explain why the amount of MeHg reaching the systemic circulation and accumulating in organs is similar in animals dosed via water or via a contaminated food matrix.

Accumulation in the organs of animals exposed to inorganic Hg is dependent on the form of administration, and is much higher when the metal is administered via drinking water than via mushrooms. Previous in vitro studies have shown the low bioaccessibility of Hg in dried mushrooms (21–40%) [2]. The authors suggest that this low Hg bioaccessibility could be due to the low digestibility of the sample or the formation of low solubility complexes of Hg with compounds present in the matrix. Chitin, one of the major long-chain carbohydrate polymers in mushrooms [36], forms strong and irreversible interactions with Hg, leading to low solubility complexes [37]. In fact, in the presence of chitin, a reduction in the bioaccessibility of inorganic Hg has been reported [2]. This would explain the low accumulation of Hg following subchronic exposure to feed enriched with mushrooms. In this case, unlike what was observed in swordfish, the strong effect of the food matrix on Hg toxicokinetics was observed.

The excretion of both Hg species reflects the accumulation profiles. Fecal elimination is higher for inorganic Hg than for MeHg, especially when Hg is administrated via drinking water. The presence of inorganic Hg in the feces of animals exposed to MeHg is worth noting, reaching a high percentage of 60–83%. This demethylation, previously evidenced in animals dosed via drinking water [38], is also observed in the present study when MeHg is conveyed through feed. This fact supports the hypothesis that the demethylation of MeHg promotes the elimination of the toxic metal [39]. Toxicokinetic studies show that MeHg is the mercury species with the highest degree of internalization, and that the fraction reaching the systemic circulation and accumulating in different organs is similar regardless of whether this mercury species is ingested via water or food. Therefore, it is essential to develop effective strategies to reduce exposure to this toxic form of Hg, with a particular focus on food as its primary source of dietary exposure.

Recently, the potential use of LAB to reduce tissue accumulation in animals exposed to Hg has been explored. Majlesi, Shekarforoush, Ghaisari, Nazifi, Sajedianfard, and Eskandari [19] reported reductions in tissue levels in animals dosed by gavage with aqueous Hg(II) solution and two LAB strains. Chen et al. [40] showed an increase in fecal excretion and reductions in hepatic accumulation in animals exposed to Hg(II) through drinking water using a daily dose of an engineered *E. coli* strain expressing the MerR protein, which possesses a mercury-binding capacity. However, the main route of exposure is through food consumption, and the predominant dietary form of Hg is MeHg. The present study is the first to evaluate the potential protective effect of LAB strains in subchronic exposures to MeHg through the diet. The LE1 and LE2 strains, isolated and characterized in vitro in this study, yielded promising results in experimental animals. The reduction in tissue contents in animals exposed via drinking water was relevant (18–64%; Figure 7A). In addition, the daily dosing of strain LE1 showed reductions in Hg contents in the internal organs of animals sub-chronically exposed through fish products (13–40%; Figure 7B). This reduction is lower than that observed in the treatments with drinking water and, as previously discussed, may be due to the chemical form in which MeHg is present in both matrices. This can modify its interaction with LAB strains in the lumen, prior to absorption. In fact, this effect is already evidenced in vitro, where the uptake of Hg by bacteria is higher in the digestion experiments with MeHg solutions (Figure 5) compared to swordfish (Figure 6).

The protective effect of the bacterial strains may be partly due to their ability to bind mercurial species [41]. The chelation of MeHg by LAB is a complex process in which cell envelope components play a relevant role. The LAB cell envelope includes peptidoglycan, teichoic acid, proteins, and polysaccharides, which contain negatively charged carboxyl, hydroxyl, and phosphoryl groups capable of binding heavy metal cations. Notwithstanding, sulfhydryl groups of proteins possibly constitute the primary chemical targets for Hg(II) and MeHg in cells [42]. The chelation of ingested Hg in the lumen may reduce the entry of the metal into the systemic circulation and tissue accumulation or may reduce the enterohepatic circulation of MeHg by preventing its reabsorption.

Moreover, LAB possibly enable other mechanisms that diminish MeHg assimilation. A recent study carried out under the same exposure conditions used in this study has shown that subchronic exposure to MeHg through drinking water causes significant disturbances in several components of the intestinal barrier, and an increase in mucosal permeability [43]. Rodríguez-Viso et al. [44] demonstrated that the bacteria used in this study, particularly strain LE1, exert a protective effect against MeHg toxic effects on the epithelium, partially restoring the disruption of the intestinal barrier. This partial restoration of the components of the barrier could also affect the intestinal transport of Hg and its subsequent tissue accumulation. Furthermore, the results reported by Rodríguez-Viso et al. [45], combined with those described in the present article, indicate that this LAB-based strategy could have a local protective effect at the intestinal level as well as a systemic effect, reducing MeHg uptake by the target organs.

## 5. Conclusions

The data obtained in the present study show that the ingestion of MeHg through food generates significant tissue accumulation, with a distribution similar to that observed when MeHg is conveyed through drinking water. In this case, the toxicokinetics of Hg are little affected by the food matrix. However, the food matrix has the potential to modify the absorption and accumulation processes, as observed for Hg(II) when it was conveyed through water or through feed prepared with mushrooms. This effect can be attributed to the presence of specific components in the food matrix (e.g., insoluble fiber) and/or the lower digestibility of this specific food. Administration of the selected LAB strains led to significant reductions in MeHg tissue accumulations. The results obtained with *L. intestinalis* LE1 suggest that LAB could be a promising strategy in combating MeHg tissue accumulation when ingested through food. Further in vivo studies, employing this strategy in animals dosed with various contaminated food matrices, are needed to support the potential use of LAB in populations chronically exposed to MeHg through the diet. However, any potential translational implications for human populations chronically exposed to MeHg through the diet should be interpreted with the appropriate caution, as the present findings are based on controlled experimental animal studies. Further in vivo investigations are required to better characterize the robustness and applicability of this approach under more complex exposure scenarios, and to support any potential extrapolation to human exposure conditions.

## Figures and Tables

**Table 1:** Mercury dose administered to rodents via water or feed. Values expressed as mg/kg body weight per day.

Treatment		Mice	Rats
Water	MeHg	0.25	0.25
	Hg(II)	0.5	0.5
Feed	Swordfish	0.25	0.024
	Mushroom	0.25	0.037

**Table 2:** Fecal concentration of total and inorganic Hg in animals exposed for 20 days to Hg via water or feed. Values expressed as ng/g and as percentages of inorganic Hg to total Hg (mean ± SD, *n* = 3–5).

Treatment		Total Hg	Inorganic Hg	Inorganic Hg (%)
Drinking water				
MeHg	Mice	1783 ± 376	1183 ± 294	67 ± 11
	Rats	3175 ± 26	2549 ± 461	83 ± 11
Hg(II)	Mice	5533 ± 1161	5086 ± 1292	91 ± 7
	Rats	18,727 ± 472	16,990 ± 551	91 ± 1
Feed				
Swordfish	Mice	1654 ± 402	1016 ± 191	60 ± 5
	Rats	488 ± 100	288 ± 71	60 ± 7
Mushroom	Mice	1808 ± 130	1526 ± 217	85 ± 8
	Rats	1142	667	61

## Data Availability

The original contributions presented in this study are included in the article/Supplementary Material. Further inquiries can be directed to the corresponding author.

## Supplementary Materials

The following supporting information can be downloaded at: https://www.mdpi.com/article/10.3390/jox16030107/s1, Table S1: Treatment groups in intervention assays with lactic acid bacteria.
