# Supplementary material for: Lactic Acid Bacteria as In Vivo Protective Strategy Against Dietary Methylmercury Exposure
Source: J Xenobiot. 2026 Jun 8;16(3):107. doi: 10.3390/jox16030107 (PMC13302665; doi:10.3390/jox16030107)
Supplement: Supplementary file 1 [file jox-16-00107-s001.zip › jox-4267206-supplementary.pdf]

Supplementary Materials: Lactic Acid Bacteria as In Vivo Protective Strategy Against Dietary Methylmercury Exposure

Luzmila Burbano, Pilar Rodríguez-Viso, Manuel Zúñiga, Vicente Monedero, Vicenta Devesa and Dinoraz Vélez

Table S1. Treatment groups in intervention assays with lactic acid bacteria

Group name	Acclimatization	Treatment
Control A	PBS (gavage)	MeHg-contaminated water, w/o bacteria (daily PBS
Group B	LE1 (gavage)	MeHg-contaminated water, daily LE1 gavage
Group C	LE2 (gavage)	MeHg-contaminated water, daily LE2 gavage
Control D	PBS (gavage)	Swordfish-containing feed, w/o bacteria (daily PBS
Grupo E	LE1 (gavage)	Swordfish-containing feed, daily LE1 gavage
Grupo F	LE2 (gavage)	Swordfish-containing feed, daily LE2 gavage
